# Personal fulfilment, sustainable working conditions and flexible employment prospects as resources for nurses’ well-being at work: a qualitative study

**DOI:** 10.1177/17449871261446832

**Published:** 2026-06-25

**Authors:** Jenni Mäntynen, Marja Hult, Terhi Saaranen

**Affiliations:** PhD Student, Department of Nursing Science, University of Eastern Finland, Finland; Psychiatry, Helsinki University Hospital and University of Helsinki, Finland; University Lecturer, Docent, PhD, Department of Nursing Science, University of Turku, Finland; Professor, Department of Nursing Science and Health Pedagogy, University of Eastern Finland, Finland

**Keywords:** employment relationships, health care organisations, nurse, well-being at work, working community

## Abstract

**Background:**

Nurse shortages are intensified by retirement and early career exits. Well-being at work is critical for retention, yet research has primarily focused on burdensome factors at work: stress and burnout. Positive aspects of well-being and the role of employment relationships remain underexplored.

**Aim:**

To describe nurses’ experiences of resources that promote well-being at work.

**Methods:**

A qualitative interview study was conducted in Finland in 2021. Seventeen nurses participated in individual interviews, and data were analysed using content analysis.

**Results:**

Resources enhancing well-being at work were grouped into four main categories: Professional fulfilment and growth, Supportive and sustainable working conditions, Organisational resources and culture and Employment flexibility. These categories included 11 subcategories, reflecting factors such as opportunities for career advancement, moderate workload, benefits and flexible employment contracts.

**Conclusions:**

Nurses’ well-being at work can be supported at individual, working community and organisational levels. Policies should promote flexible employment relationships, enable professional development and strengthen organisational practices that foster well-being. Legislative frameworks and organisational strategies are essential to improve retention and address workforce sustainability in nursing.

## Introduction

Well-being at work is a key factor influencing nurses’ intention to remain in the profession (Bae, 2023; Galbany-Estragués et al., 2024). While reasons for leaving nursing and strategies to improve the sector’s attractiveness have been widely studied (Al Zamel et al., 2020; Maniscalco et al., 2024; Marufu et al., 2021), existing research has largely emphasised negative aspects such as stress, workload and burnout (Albalawi et al., 2024; Alves et al., 2023). As a result, considerably less is known about positive resources that promote nurses’ well-being at work. There is a growing need to understand these constructive elements, as they play a central role in strengthening retention. Previous studies highlight the importance of adequate staffing, supportive leadership, continuous education and interdisciplinary collaboration (Bae, 2023; Galbany-Estragués et al., 2024), and working conditions consistently emerge as the most influential factor for retention.

Globally, the demand for nursing staff continues to rise as populations age and multimorbidity becomes more common (De Vries et al., 2024; Matsuo et al., 2021). At the same time, workforce shortages persist, with many vacancies unfilled (Marufu et al., 2021; Medore et al., 2024). Intent to leave is a worldwide concern: approximately 9% of nurses plan to exit the profession, often within a few years of graduation. By 2030, the shortage is projected to reach 4.8 million nurses and midwives (Chen et al., 2024; World Health Organization (WHO) 2024). In ageing societies, retirements and insufficient inflow of younger nurses further worsen the problem (Medore et al., 2024).

Well-being at work is conceptualised across individual, group and organisational levels (Antoniadou et al., 2023). Organisations that invest in employee well-being benefit from higher productivity, stronger commitment and improved retention (Hussain et al., 2022). Key dimensions of well-being include job content, collegial relationships, supervision, compensation, benefits, communication and career clarity. Several models address workplace well-being (Gelencsér et al., 2023). One practical framework highlights four aspects: working conditions, professional competence, working community and workers’ characteristics, including workload and work requirements (Laine, 2018; Saaranen et al., 2015). Additionally, the Job Demands-Resources (JD-R) model conceptualises well-being as the result of a balance between job demands and job resources, proposing that resources not only buffer the negative effects of high demands but also promote motivation, engagement and well-being. Job resources may operate at multiple levels, including individual autonomy, social support within teams and organisational practices and structures (Demerouti et al., 2001). This framework provides a useful lens for examining how different types of resources contribute to nurses’ well-being at work and is further elaborated in relation to study findings in the Discussion section. However, private life factors also influence well-being at work (Kärkkäinen et al., 2019).

One important yet underexplored area concerns the role of employment relationships in promoting nurses’ well-being at work. The evidence suggests that flexible schedules and shift arrangements positively affect well-being at work (Galbany-Estragués et al., 2024), and younger generations in particular value diverse contract types and ability to balance work with personal life (Nunstedt et al., 2020; Wardana et al., 2020). In Nordic countries, for example, voluntary fixed-term and agency work have increased; one-fifth of Finnish nurses hold fixed-term positions (Findicator, 2021; Ilsøe and Larsen, 2021). Temporary arrangements may offer flexibility, higher hourly wages and autonomy (Gahrmann and Klumb, 2024; Hult et al., 2022). Although employment relationships are typically categorised as permanent or fixed-term (Fu et al., 2023; Senek et al., 2020), they also include agency work, student placements, apprenticeships, family leave, part-time work and phased retirement. However, little is known about how nurses experience these diverse arrangements as potential for well-being. Some studies even suggest that temporary workers report higher job satisfaction than permanent staff (Hult et al., 2022; Vainieri et al., 2019), indicating a need for deeper understanding.

Despite extensive research on nurse well-being and retention, important knowledge gaps persist. Previous studies have rarely examined well-being from a perspective of positive, multilevel resources, nor have they sufficiently explored the role of employment flexibility as a well-being factor. To address these gaps, this study explores nurses’ experiences of resources that promote well-being at work across individual, community and organisational levels. By focusing on the positive dimension of well-being and the role of employment flexibility, the study aims to generate new insights that can inform workforce development and support nurse retention.

## Aim

The aim of this study was to describe nurses’ experiences of resources that promote well-being at work.

## Methodology

### Study design

We conducted a qualitative interview study to gain an in-depth understanding of nurses’ subjective experiences of work-related well-being, which aligns with the exploratory nature of the research questions (Streubert and Carpenter, 2011). This approach allowed participants to articulate personal meanings and contextual factors.

### Study participants

Participants were recruited in spring 2021 as part of a broader survey on employment quality in the social and healthcare sectors, conducted in collaboration with Finnish trade unions and a staff leasing company. At the end of the survey, respondents were invited to indicate their interest in participating in a qualitative interview study by voluntarily providing their email address. Only those nurses who explicitly expressed interest in further participation were contacted by the research team. From this group, registered nurses were purposively selected for the present study, as the research focused specifically on registered nurses’ experiences. A total of 35 registered nurses who met the inclusion criteria and had provided their contact details were approached by email with written information about the study purpose, voluntary nature of participation and confidentiality. Participation was entirely voluntary, no incentives were offered, and recruitment was conducted independently of employers to avoid any potential coercion. Of the 35 nurses invited, 17 consented to participate and were included in the study. Participants varied in age, professional experience, employment status and organisational context, including primary and specialised care, enhancing variation in data and supporting transferability.

### Data collection

After obtaining informed consent and permission to record the interviews, the data were collected through semi-structured interviews. The interview guide was based on previous research (Kallio et al., 2016; Turner, 2010), and the interviews were conducted remotely via Microsoft Teams. The interview guide is provided as Supplemental Material 1. Data collection continued until no new subcategory properties emerged; saturation was reached after 15 interviews. However, all 17 nurses who had signed up were interviewed. The interviews were audio-recorded and lasted between 19 and 41 minutes, with an average duration of 30 minutes, resulting in a total of 8.3 hours. The first author transcribed the interviews verbatim in Finnish using Microsoft Word. During each interview, notes were taken regarding thoughts, wording and observations. The resulting material comprised 83 pages, formatted in Times New Roman, 12-point font, with 1.5 spacing. The categorisation process was conducted in Microsoft Excel, where the data were subsequently translated into English.

### Data analysis

Data were analysed using inductive content analysis (Elo and Kyngäs, 2008; Lindgren et al., 2020). The researcher first read the transcripts repeatedly to gain a comprehensive understanding of the nurses’ experiences. Meaningful units, which were sentences or coherent units of thought, were then identified and extracted. The units were condensed and documented in Microsoft Excel, after which they were grouped based on similarities and differences. Similar expressions were combined into subcategories and given descriptive names. During the abstraction process, subcategories with related content were merged into broader categories. Regular discussions with the research team strengthened dependability and minimised researcher bias. The analysis resulted in the formation of four categories reflecting the core findings of the study (Elo and Kyngäs, 2008; Lindgren et al., 2020;).

### Ethical considerations

The study adhered to established principles of reliability, honesty, respect and accountability (ALLEA, 2023). Research permits were obtained from the Institutional Review Boards of the participating trade unions and the staff leasing company. The study followed national guidelines for responsible conduct of research, and all participants provided informed consent (Finnish Advisory Board on Research Integrity, 2019). Invitations to participate were distributed via email and included detailed information and consent forms. Participants were informed that interviews would be recorded and used solely for research purpose, and participation was voluntary. All identifying information was removed during the analysis to ensure confidentiality and to prevent the identification of individual participants. The reporting of this qualitative study was guided by the Standards for Reporting Qualitative Research (SRQR) checklist (O’Brien et al., 2014), which is provided as Supplemental Material 2.

Trustworthiness was ensured in accordance with the criteria by Lincoln and Guba (1985). Credibility was supported through the use of a semi-structured interview guide, prolonged engagement with the data and verbatim transcription of interviews. Dependability was enhanced through a systematic and transparently documented research process, including detailed descriptions of the data collection and analysis procedures. Confirmability was strengthened through careful documentation of analytic decisions and the use of researcher reflexive notes during data collection and analysis. Transferability was supported by providing detailed description of the study context, participants and data, enabling readers to assess the applicability of the findings to other settings.

## Results

### Participants’ characteristics

All participants were registered nurses (*n* = 17) with professional qualifications, eight of whom held a master’s degree. The sample consisted of clinical nurses (*n* = 11), a public health nurse (*n* = 1), managers (*n* = 3) and clinical specialists (*n* = 2). All participants were women aged 28–60 years, with an average age of 48. Their work experience ranged from 2.5 to 36 years, with an average of 18.5 years. Sixteen nurses held permanent employment contracts, and one had a fixed-term contract. One participant worked part-time.

**Figure 1.**
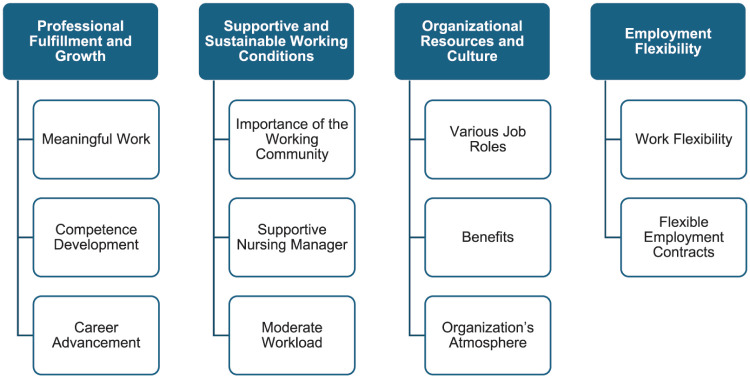
Four main categories and eleven subcategories presenting the results of the study.

The findings related to resources that enhance well-being at work were organised into four main themes: Professional fulfilment and growth, Supportive and sustainable working conditions, Organisational resources and culture and Employment flexibility. Figure 1 presents the main categories and their subcategories.

### Professional fulfilment and growth

#### Meaningful work

Nurses described meaningful work as a central resource for well-being. A clear job description, opportunities to assume diverse roles and organisational support for role development were seen as key contributors. Role variation, such as working across multiple units or engaging in rotational assignments, introduced diversity and a dynamic workflow, which nurses found motivating. A well-defined and meaningful job description was perceived to facilitate professional development and enhance nurses’ influence over their work.

Experiencing meaningful work also strengthened nurses’ expectations and commitment to the profession. Expectations differed across age groups, with younger nurses expressing higher demands regarding working conditions and organisational support. Opportunities for professional growth were viewed as particularly valuable, fulfilling personal needs and reinforcing commitment. Several nurses noted that their sense of calling had diminished over time; work was no longer seen as a lifelong mission, but as a profession requiring balance.

The new generation has entered the industry; they have very different expectations of work than the old generation. (Nurse 11)

#### Competence development

Nurses emphasised the importance of comprehensive orientation for newly hired employees, noting that the process had improved in response to their feedback. Effective orientation was considered essential for enabling new nurses to succeed in their roles. The transfer of tacit knowledge from experienced nurses to newcomers was highlighted as critical and working in senior–junior pairs was described as an effective mechanism for skill transmission. Nurses also valued responsibility-based work and expressed a strong desire for continuous learning and further education. Beyond routine tasks, participation in unit-level quality improvement activities was regarded as meaningful.

Senior nurses have a lot of tacit knowledge, and there is a danger that tacit knowledge will not be distributed to new nurses in a high-speed environment. (Nurse 7)

#### Career advancement

Nurses considered opportunities for career development highly relevant, particularly noting that younger generations expected clearly defined career paths. They expressed interest in career options that extend traditional managerial or clinical expert roles, such as specialist nurse, development nurse or teaching nurse positions. Career advancement models were perceived as enhancing competence and influencing compensation. Nurses also stressed the need for organisational support for educational pursuits, including the provision of study time during working hours.

There are different careers; some may wish to take on a leadership role, while others prefer to take on a specialist position; it is also possible, hopefully, to stay directly involved in patient care. (Nurse 17)

### Supportive and sustainable working conditions

#### Importance of a working community

Nurses reported that well-being was strengthened through teamwork, mutual support, trust, equality and a strong sense of community. Smooth workflows, shared goals and the ability to meet work-related expectations were described as rewarding. Collaboration fostered solidarity, while supervision, departmental meetings and team-building activities were considered beneficial. Nurses depicted their workplaces as open, humorous, dialogical environments that supported a sense of belonging. A positive team spirit was seen as reducing stress, and nurses emphasised that individual attitudes and behaviours influence the entire working community. They also acknowledged shared responsibility for maintaining well-being at work.

Then the working atmosphere is very nice, and it is not bad to go work every day. Coworkers play an important role. (Nurse 1)

#### Supportive nursing manager

Nurses underscored the critical role of nursing managers in shaping the work environment and influencing employment choices. They valued managers who demonstrated genuine interest in staff well-being, responded to concerns and were familiar with their teams. Leadership qualities such as fairness, equality and accountability were considered essential. The physical presence, accessibility and responsiveness of the unit manager were especially appreciated. A supportive manager who actively engaged with staff was viewed as a key contributor to a positive workplace climate.

Equality and transparency are an important part of work well-being. We all want to be seen, encouraged, and appreciated. (Nurse 7)

#### Moderate workload

Nurses emphasised that maintaining adequate staffing levels, temporarily reducing patient beds when necessary and ensuring sufficient resources were crucial for achieving a manageable workload. An appropriate workload was seen as essential for providing high-quality and safe patient care. A humane workload also depended on mutual support among colleagues.

You can do your work so that you don’t have a bad conscience that you are too busy. (Nurse 1)

### Organisational resources and culture

#### Various job roles

Nurses highlighted the importance of enabling diverse job roles within the organisation. Potential employees included students, retirees and individuals with partial work capacity. They noted that adequate guidance, orientation and workplace learning opportunities were essential for students’ successful integration. For employees with physical limitations, adapting job descriptions and modifying tasks were considered necessary to maintain engagement. Nurses also suggested offering alternative work arrangements during sick leave to support productivity and connection to the workplace.

A phone service has been built for the emergency room, so there were nurses like those who could not do the work that involves physical strain. So, they could be in the phone service; experienced nurses have been obtained, and they have found the work meaningful. (Nurse 17)

Nurses further proposed reconsidering which professional groups could contribute to nursing work. Suggestions included integrating apprentice students, implementing job-sharing models, outsourcing selected tasks and introducing new professional roles within care teams.

Nursing is changing, and we should be involved in it—not only with this training and competence but also with people who have other trainings that will be ready for health care. (Nurse 14)

#### Benefits

Nurses viewed fair compensation as a central element of feeling valued at work. They expected wages to correspond with job demands, competence and career progression, emphasising that salary adjustments should follow increases in skills and responsibilities. Several experienced nurses noted that salary development often plateaued after roughly ten years, which influenced considerations about changing positions. Financial recognition was seen as important, but nurses also hoped for acknowledgement for achievements through symbolic gestures and celebrations. In addition, practical benefits during working hours, such as subsidised meals and the provision of coffee were appreciated.

Of course, there is also salary that gives you the feeling of appreciation. (Nurse 10)

Employer–provided benefits, including sports and cultural vouchers, recreation funds and organised events, were received positively. Nurses expressed interest in additional opportunities to support well-being, such as access to a workplace fitness area, hobby groups or the possibility to exercise during working hours. Campaigns that promoted physical activity and social interaction, for instance by encouraging employees to find a jogging partner, were also viewed favourably.

In general, it should be automatic to support well-being at work, such as cultural and sports vouchers. (Nurse 15)

#### Organisation’s atmosphere

Nurses described work-related well-being as shaped by individual factors as well as team and organisational practices. For younger generations, organisational commitment to well-being was considered an important recruitment factor. Participants expressed trust that organisation intervened when issues arose and emphasised the need for comprehensive occupational health services. Although individuals are responsible for maintaining their own health, timely access to support was seen as essential.

The well-being at work will become a serious problem if the employers do not recognize how to deal with it. The future will look dark. (Nurse 11)

A relatively flat decision-making structure was perceived to enhance influence and participation in workplace matters. Respectful interaction between professional groups and organisational flexibility were described as important contributors to positive atmosphere. During major organisational changes, nurses hoped to be genuinely consulted and for their perspectives to be reflected in decisions.

Well-being at work must be so that I can also influence flexibility and negotiation opportunities, and I can say my opinion and be heard. (Nurse 8)

Nurses highlighted the significance of an organisation’s reputation in both recruitment and retention. The image communicated to prospective employees, through effective onboarding, clear communication and opportunities for professional development, was considered highly influential. They also hoped that everyday working conditions, collaboration and the meaningful work would be emphasised. According to participants, organisations that responded well to staff expectations tended to have better retention outcomes, and strategic investments in marketing and recruitment could strengthen the ability to attract new nurses.

The most important factor is the reputation. What image has been created of employers and the workplace? How is it implemented at work, and how are these promises redeemed? (Nurse 17)

### Employment flexibility

#### Work flexibility

Nurses reported that remote work opportunities increased their overall sense of flexibility, enabling a more effective balance between professional and family responsibilities. Remote arrangements allowed certain tasks to be completed from home, and ward nurses expressed interest in participating in training sessions or completing online courses remotely. Nurses also emphasised that involvement in shift planning enhanced their control over work schedules and contributed positively to well-being at work. Collaborative shift scheduling was considered particularly beneficial, as it accounted for individual preferences; some nurses preferred night shifts, whereas others favoured daytime work. Moreover, shift planning was seen as a practical mechanism for accommodating ongoing educational needs.

We have collaborative work shift planning; it supports well-being at work because you can affect your own schedule. (Nurse 11)

Nurses further highlighted the importance of maintaining a healthy balance between work and leisure time. They hoped that employment arrangements would be adaptable to different stages of life, allowing, for example, those on parental leave or approaching retirement to work selectively. Flexible arrangements such as part-time schedules, morning shifts and adjustable work–hour percentages were highly valued. Leisure time was viewed as essential for recovery, with restorative effects that enhanced both well-being and enthusiasm in the workplace.

It’s part of our life, and we have a life outside of work, which is great. (Nurse 8)

#### Flexible employment contracts

Nurses observed that employment relationships had become shorter, a change perceived positively because it allowed them to gain experience in different organisations. Younger nurses no longer expected to remain in the same workplace until retirement. Attractive job opportunities were associated with diverse tasks and prospects for professional growth.

I think employment relationships will become shorter than they were in the past. Then there are no more people who have been working in the same place for 15 years. (Nurse 1)

Nurses noted an acceleration in turnover, with more frequent job changes than before. A plentiful supply of available positions provided nurses with greater freedom to choose where to work. Decisions to change jobs were influenced by factors such as job satisfaction, the nature of the work tasks and personal life circumstances. Even nurses holding permanent contracts were now more likely to change employers compared to previous years.

People change places. It is not necessarily good to reach a pension position. (Nurse 14)

Temporary employment contracts were described as historically common in healthcare. For some nurses, permanent employment was not a priority; instead, they preferred fixed-term arrangements to avoid long-term commitment and to maintain flexibility. Temporary contracts also enabled exposure to various work environments.

Some nurses seem to like being temporary workers; they don’t necessarily dare to commit if they want [to] do something else. (Nurse 10)

However, several nurses expressed a preference for long-term employment due to stability and benefits associated with permanent contracts. This perspective was partly linked to experiences during the economic recession of the 1990s. In addition, some nurses desired continuity and expected to remain in one workplace throughout their careers.

We generally want permanent posts, because there are more possibilities to influence your own work. (Nurse 9)

Nurses emphasised the need for employment relationships to adapt to individual life circumstances. Some preferred temporary contracts because they offered flexibility in shift selection and opportunities for higher pay. Although freelance work had previously been associated with limited job employment prospects, the current abundance of available positions enables nurses to choose their preferred workplaces. Seasonal work was also considered attractive, particularly among younger nurses who wished to work part of the year and travel abroad. Generational differences in employment preferences were evident: younger nurses frequently changed jobs or organisations and favoured short-term contracts immediately after graduation. Permanent employment gained importance later in life, especially when starting a family.

You can see there are a lot of young people who basically start from the idea they will work here for half a year and then go out into the world for half a year. (Nurse 17)

## Discussion

This qualitative study identifies four interrelated categories that promote nurses’ well-being at work: Professional fulfilment and growth, Supportive and sustainable working conditions, Organisational resources and culture and Employment flexibility and shows that well-being emerges from their combined, multilevel effects at individual, communal and organisational levels. A central contribution concerns employment flexibility, which surfaces as the most distinctive finding and helps explain how contemporary nursing work can better accommodate diverse life circumstances while supporting engagement and retention.

Professional fulfilment and growth support well-being at work when role expectations are clear, work is meaningful and opportunities for learning and career progression are credible and accessible. This aligns with prior evidence linking meaningful work and development pathways to stronger commitment and professional growth and to lower intentions to leave (Alves et al., 2023; Maniscalco et al., 2024; Muir et al., 2024).

Support from colleagues and managers also plays a key role. Nurses value an open, trusting and supportive community, whereas effective communication and collaborative problem solving help maintain a positive atmosphere (Mirzaei et al., 2024). Sustainable working conditions, including manageable workloads, adequate staffing and flexible scheduling, are essential for preventing strain and maintaining work–life balance. Earlier studies confirm the strong influence of workload and resourcing on well-being (Alves et al., 2023; Hahm et al., 2024; Masanotti et al., 2020) as well as the importance of social support from both colleagues and managers (De Vries et al., 2023; Hahm et al., 2024; Maniscalco et al., 2024).

Organisational resources and culture strongly influence nurses’ well-being. Nurses expect fair compensation that reflects the demands and responsibilities of the role, supplement by benefits such as wellness services, recreational opportunities and accessible occupational health support. Previous research highlights salary, benefits and workplace facilities as important sources of motivation (Albalawi et al., 2024; Alves et al., 2023; Hahm et al., 2024). Organisation’s reputation, shapes by high-quality onboarding, transparent communication, favourable working conditions and meaningful work, also affects well-being and retention. Research shows that strong organisational commitment reduces turnover and supports long-term retention (De Vries et al., 2024).

Generational differences also shape experiences of well-being. Younger nurses place greater emphasis on a positive organisational atmosphere and opportunities for growth, while collaboration between junior and senior nurses supports mutual learning. Supportive work environments promote job embeddedness among new nurses. Providing learning opportunities fosters belonging and helps new nurses adapt, especially when workplace practices align with their values (Fan et al., 2024).

Employment flexibility is where this study most clearly extends current understanding. Nurses value a spectrum of options: permanent, fixed-terms, part-time, seasonal or on-call and the capacity to adjust this over time as life circumstances change. Younger nurses may prefer varied experiences and shorter commitments, whereas permanent contracts continue to provide stability for those with family responsibilities. Fixed-term contracts offer flexibility, access to the labour market and opportunities to balance work with study or travel (Gevaert et al., 2023; Vainieri et al., 2019). Flexible scheduling (including collaborative shift planning) and the possibility of extended breaks beyond statutory leave also support well-being and engagement. Previous research emphasises that work–life balance should extend beyond family-related events to benefit all employees (Matsuo et al., 2021).

Within the JD-R framework, flexibility functions as a job resource that enhances autonomy, supports recovery and helps buffer strain under high demands (Bakker and Demerouti, 2014). Our findings extend this framework by demonstrating that flexibility operates simultaneously at multiple levels, including the individual (choice of contract), team (collaborative shift planning) and organisational levels (availability of alternative employment pathways), a perspective that has received limited attention in previous nursing research.

Despite its benefits, flexibility also introduces potential tension that organisations must anticipate and manage. Heavy reliance on temporary or short-terms contracts can challenge continuity of care and weaken team cohesion unless role boundaries, handover protocols and knowledge-transfer mechanisms are robust (Senek et al., 2020; Vainieri et al., 2019). Rapid career mobility and frequent transitions, while enriching for individuals, may complicate equitable workload distribution and unit-level stability. These tensions do not negate the benefits of flexibility; rather, they underscore the need for targeted implementation. This includes maintaining a core staffing baseline, adding flexible capacity to manage variability, codifying fair, team-based scheduling principles, and integrating structured mentoring and orientation practices to safeguard patient safety as teams adapt (Lapalme and Guerrero, 2019; Muir et al., 2024).

Bringing the four main categories together points to a systems’ view of well-being. Professional fulfilment and growth, supportive and sustainable working conditions, organisational resources and culture and employment flexibility – illustrate that nurses’ well-being arises from interplay of multiple, mutually reinforcing factors. Meaningful work and opportunities for development strengthen nurses’ sense of purpose, whereas supportive teams and fair organisational practices provide the stability and structure for coping with daily demands. Employment flexibility complements these elements by granting autonomy and allowing work arrangements to adapt to life circumstances. When combined, these resources create a coherent system in which personal growth, supportive relationships, organisational fairness and flexible structures jointly sustain well-being and enhance retention. This integrated perspective highlights that well-being cannot be improved through single interventions but requires coordinated action across multiple levels of the work environment.

Although several findings align with established research on nurse well-being, this study provides distinct and meaningful contributions. Firstly, it conceptualises employment flexibility not only as a determinant of turnover but also as a positive, multidimensional resource when implemented with equity and safeguards. Secondly, it integrates individual-, team- and organisational-level resources into a single explanatory framework, clarifying how their interaction sustains well-being and supports retention. Thirdly, it highlights generational differences in how resources are valued, particularly opportunities for growth and work-life flexibility, which can inform the development of targeted retention strategies for diverse nursing workforce.

This study was conducted within the Finnish and Nordic labour market context, characterised by publicly funded healthcare, strong labour legislation and high unionisation. Consequently, the prominence of comprehensive occupational health services, structured career pathways in public organisations and employment routes are partly context-specific (Ministry of Social Affairs and Health, 2024a; 2024b). Nevertheless, many implications are transferable. Meaningful work, supportive leadership, manageable workloads, transparent communication and opportunities for professional development are widely recognised as key drivers of nurses’ well-being and retention internationally. Flexibility can also enhance well-being in other systems, provided it is balanced with continuity, fairness and patient safety.

### Strengths and limitations

A qualitative interview approach was chosen to gain nuanced perspectives on the phenomenon under study. The sampling strategy targeted nurses with first-hand experience of the topic, enabling the collection of rich and relevant data. Although the number of participants was relatively small and interview lengths varied, consistent themes emerged across cases, indicating data saturation. All participants were women, reflecting the highly gendered nature of nursing profession in Finland. Although this limits the representation of other gender perspectives, the sample included nurses from different geographical regions, organisational settings and employment forms, which can be considered a strength of the study. Recruitment through professional unions, covering approximately 80% of Finnish nurses, further supported access to participants with diverse professional backgrounds.

### Implications

The findings of this study have implications for nursing practice, education, healthcare systems and policy and future research. The results underscore the importance of flexibility, meaningful work and supportive working conditions as key resources for nurses’ well-being. Nursing leaders should consider how flexible employment arrangements and collaborative work practices can enhance autonomy, recovery and retention for sustainable nursing careers. Nursing education should prepare students to navigate diverse employment relationships and recognise factors that support well-being at work. Integrating content on career planning, employment forms and self-care may help nurses sustain their well-being across different career stages. At the system level, flexibility and supportive employment structures should be recognised as resources for workforce sustainability. Policies that enable flexibility while maintaining job security may improve the attractiveness of nursing and support long-term workforce stability. Future studies should examine the long-term effects of flexible employment arrangements on nurses’ well-being across different contexts and populations, using longitudinal and comparative designs.

## Conclusion

This study highlights key resources that support nurses’ well-being at work across individual, working community and organisational levels. In addition to meaningful work, professional development, supportive leadership and sustainable working conditions, the findings offer novel insights by identifying employment flexibility as a central resource for well-being. Importantly, the study extends knowledge on work well-being by demonstrating that flexibility operates as a job resource at multiple levels, including individual choices regarding employment contracts, team-level practices and organisational employment structures. Flexibility is thus not limited to scheduling or work–life balance but encompasses diverse contract types, career mobility and changing employment preferences, particularly among younger generations. These findings call for healthcare organisations and policymakers to align employment policies with evolving workforce expectations. Supporting nurses’ well-being through flexible and sustainable employment practices can strengthen retention and enhance the long-term attractiveness of the nursing profession.

**Key points for policy, practice and research**

- Flexibility in nursing extends beyond work schedules to include employment contracts, job types and contract duration, reflecting a growing group of nurses seeking short-term, varied and mobile career pathways.
- Nurses’ well-being at work is supported by resources operating at individual, community and organisational levels, highlighting the need for multilevel interventions.
- Nurses’ expectations regarding work and careers are evolving, with increasing emphasis on flexibility and mobility across the career span, requiring organisational responsiveness.

## Supplemental Material

sj-docx-1-jrn-10.1177_17449871261446832 – Supplemental material for Personal fulfilment, sustainable working conditions and flexible employment prospects as resources for nurses’ well-being at work: a qualitative study Supplemental material, sj-docx-1-jrn-10.1177_17449871261446832 for Personal fulfilment, sustainable working conditions and flexible employment prospects as resources for nurses’ well-being at work: a qualitative study by Jenni Mäntynen, Marja Hult and Terhi Saaranen in Journal of Research in Nursing

sj-docx-2-jrn-10.1177_17449871261446832 – Supplemental material for Personal fulfilment, sustainable working conditions and flexible employment prospects as resources for nurses’ well-being at work: a qualitative study Supplemental material, sj-docx-2-jrn-10.1177_17449871261446832 for Personal fulfilment, sustainable working conditions and flexible employment prospects as resources for nurses’ well-being at work: a qualitative study by Jenni Mäntynen, Marja Hult and Terhi Saaranen in Journal of Research in Nursing
