# Supplementary material for: Personal fulfilment, sustainable working conditions and flexible employment prospects as resources for nurses’ well-being at work: a qualitative study
Source: J Res Nurs. 2026 Jun 25:17449871261446832. Online ahead of print. doi: 10.1177/17449871261446832 (PMC13309349; doi:10.1177/17449871261446832)
Supplement: sj-docx-1-jrn-10.1177_17449871261446832 – Supplemental material for Personal fulfilment, sustainable working conditions and flexible employment prospects as resources for nurses’ well-being at work: a qualitative study [file sj-docx-1-jrn-10.1177_17449871261446832.docx]

**Supplementary Material 1. Interview Guide**

**1. Background information**

- Age
- Gender
- Place of residence
- Education
- Profession
- Type of employment contract
- Work experience (years)

**2. The relationship between work and health in nursing**

- What do you think is included in occupational health?
- What significance does health have in today´s working life?
- How is health taken into account in job seeking/ recruitment and in work tasks?
- How is health promoted at work, and whose responsibility is it?

**3. Well-being at work**

- What do you think is included in well-being at work?
- What significance does well-being at work have in today´s working life?
- How is well-being at work taken into account in job seeking/ recruitment and in work tasks?
- How is well-being at work promoted at work, and whose responsibility is it?

**4. Employment relationships of nurses**

- What kinds of employment relationships exist in healthcare, how have they changed, and why?
- What will employment relationships be like in the future, why, and what would be desirable versus likely?
- How can employment relationships support nurses´ career paths?
- How can they support health and well-being at work?

**5. Nursing now and in the future**

- What are nurses like as employees today?
- What kind of employees will be needed in nursing in the future? What characteristics should they have, and why?
